# Luteolin Inhibits Inflammatory Responses via p38/MK2/TTP-mediated mRNA Stability

**DOI:** 10.3390/molecules18078083

**Published:** 2013-07-09

**Authors:** Wanling Wu, Dongye Li, Yu Zong, Hong Zhu, Defeng Pan, Tongda Xu, Tao Wang, Tingting Wang

**Affiliations:** 1 Institute of Cardiovascular Disease Research, Xuzhou Medical College, 84 West Huaihai Road, Xuzhou 221002, Jiangsu, China; 2 Department of Cardiology, Affiliated Hospital of Xuzhou Medical College, 99 West Huaihai Road, Xuzhou 221002, Jiangsu, China

**Keywords:** luteolin, anti-inflammatory, bone marrow macrophages, mRNA stability

## Abstract

Luteolin (Lut) is a common dietary flavonoid present in Chinese herbal medicines that has been reported to have important anti-inflammatory properties. The purposes of this study were to observe the inhibition of lipopolysaccharide (LPS)-induced inflammatory responses in bone marrow macrophages (BMM) by Lut, and to examine whether this inhibition involves p38/MK2/TTP-mediated mRNA stability. Lut suppressed the production of tumor necrosis factor-α (TNF-α) and interleukin-6 (IL-6) in a dose-dependent manner according to enzyme-linked immunosorbent assay (ELISA) analysis. Lut also shortened the half-lives of the TNF-α and IL-6 mRNAs according to real-time PCR analysis. Western blots were performed to assess the activation of p38 and MK2 as well as the expression of TTP. The results indicated that Lut inhibited p38 and MK2 phosphorylation while promoting TTP expression. These results suggest that the anti-inflammatory effects of Lut are partially mediated through p38/MK2/TTP-regulated mRNA stability.

## 1. Introduction

**Figure 1:**
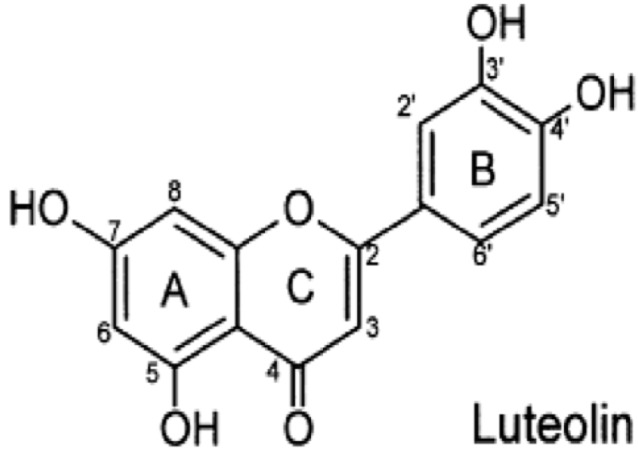
Chemical structure of luteolin (3′,4′,5,7-tetrahydroxyflavone).

Over the years, many epidemiological studies have indicated that flavonoids can reduce the incidence of chronic diseases such as cardiovascular disease and cancer [1,2]. In addition, dietary flavonoids have been shown to modulate acute and chronic inflammatory responses [3]. Luteolin (Lut, Figure 1), a common dietary flavonoid present in Chinese herbal medicines, has been reported to have important anti-inflammatory properties [4,5]. Our previous studies have demonstrated the anti-atherosclerosis (AS) activities of Lut in H_2_O_2_-induced vascular smooth muscle cells and angiotensin II (Ang II)-induced human umbilical vein endothelial cells with regards to proliferation and migration [6,7].

It is well known that chronic inflammation plays a pivotal role in the pathogenesis of AS. The recruitment of leukocytes and the expression of inflammatory cytokines are both involved in the early stages of atherogenesis [8]. Activated macrophages secrete inflammatory cytokines, such as tumor necrosis factor-α (TNF-α) and interleukin-6 (IL-6), which are key players in the promotion of vascular inflammation; these molecules amplify the local inflammatory response at the site of arterial injury. TNF-α and IL-6, which increase in abundance during AS, are used as predictive biomarkers of cardiovascular disease and cardiovascular mortality [9,10]. Thus, reducing TNF-α and IL-6 production has been suggested as an important therapeutic strategy for the prevention of AS.

The expression of inflammatory genes is known to be regulated by multiple processes, including mRNA stability [11]. In recent years, the regulation of mRNA stability has been gradually considered to be an important factor affecting the expression of these genes. The rate of mRNA degradation is related to its stability, which is determined by its own structure and internal sequence. The importance of adenine-uridine-rich elements (AREs) located in 3′-untranslated regions (3′-UTRs) is well recognized [12,13,14]. Many ARE-containing mRNAs can be stabilized in response to extracellular stimuli such as IL-10 or lipopolysaccharide (LPS) [15]. The regulatory function of AREs is mediated, in part, via the action of RNA-binding proteins, which recognize and bind the ARE sequence [16,17,18,19].

Tristetraprolin (TTP), a substrate of p38 mitogen-activated protein kinase (MAPK)-activated protein kinase 2 (MK2), is one of the RNA-binding proteins that can accelerate the degradation of ARE-containing mRNAs [20]. TTP binds to AREs located in the 3’ UTR of target mRNAs and directs them to the exosome for rapid degradation [21,22]. The TTP protein is phosphorylated by MK2 [23,24], and its stability has been reported to be regulated by the p38/MK2 pathway. The p38/MK2 pathway has been reported to be a crucial regulator of the expression, stability, and function of TTP [23,25,26]. The activation of the p38/MK2 pathway has been shown to abolish TTP-mediated repression of IL-6 3′-UTR reporter activity [27]. It has been shown that the p38/MK2 cascade is involved in regulating the mRNA stability of ARE-containing mRNAs [28]. In macrophages, the structure of the 3’-UTR ARE of IL-6 is essential to the mechanism that regulates the stability of this mRNA. The mechanism by which the TNF-α and IL-6 mRNAs are targeted by TTP has been intensely studied [27,29]. Previous studies have suggested that the TTP-mediated regulation of the mRNA stability of TNF-α and IL-6 is largely downstream of the p38/MK2/TTP signaling cascade [30].

At present, there are many studies on the anti-inflammatory effects of Lut and its pharmacological mechanisms of action [5,31]. Several mechanisms underlying the inhibition of LPS-induced inflammatory cytokine production by Lut have been investigated, such as signaling through the TRIF and MAPK pathways [5,32,33]. However, whether the anti-inflammatory properties of Lut in LPS-induced bone marrow macrophages (BMM) involve mRNA stability processes under the regulation of the p38/MK2/TTP signaling cascade is not fully appreciated. Thus, the purpose of this study was to observe the inhibition of LPS-induced inflammatory response of BMM by Lut and to elucidate whether this property is dependent on the p38/MK2/TTP signaling pathway. Furthermore, we focused our examination of the p38/MK2/TTP cascade on the post-transcriptional branches that are affected by Lut.

## 2. Results and Discussion

### 2.1. Lut Inhibits the Production of TNF-α and IL-6

**Figure 2:**
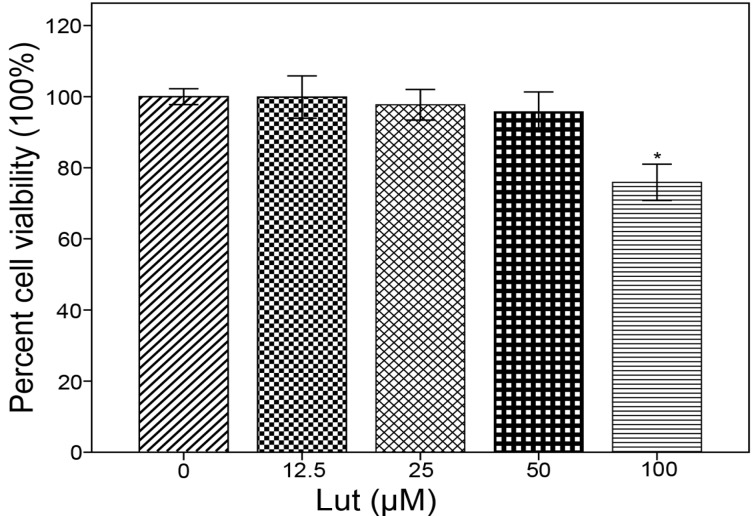
MTT assay BMM were incubated with concentrations of luteolin (12.5, 25, 50 and 100 μM) for 12 h, and then the absorbance of value of each group was measured by MTT assay as described in the methods section. Cell viability of the control group was considered to be 100%, * *p* < 0.05.

In the present study, the anti-inflammatory effects of Lut in LPS-induced BMM were examined. BMM viability in the presence of various concentrations of Lut (12.5–100 μM) was measured by the 3-(4,5-dimethyl-2-thiazyl)-2,5-diphenyl-2*H*-tetrazolium bromide (MTT) reduction assay. The results showed that Lut, at concentrations of 12.5, 25 and 50 μM, did not affect the viability of BMM; however, BMM viability was negatively affected by 100 μM Lut (Figure 2). Therefore, for the further studies, Lut concentrations of 12.5, 25 and 50 were employed.

**Figure 3:**
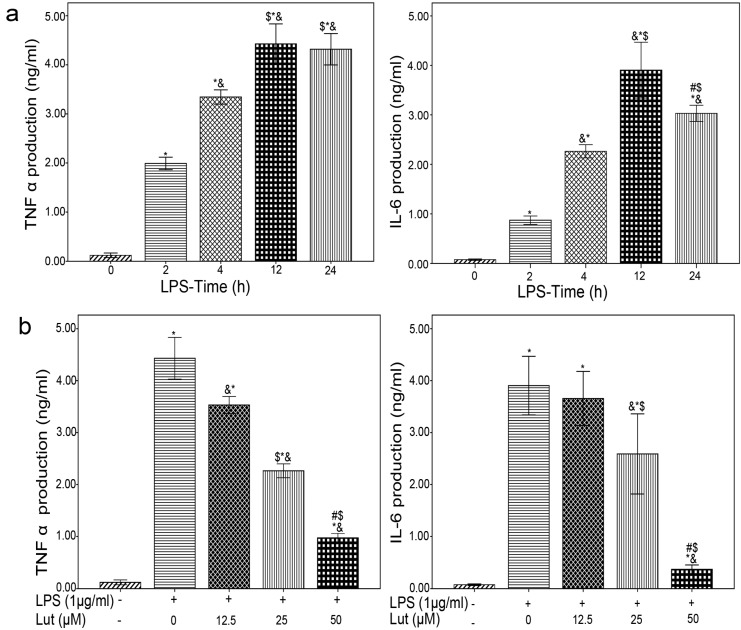
Lut inhibits the production of TNF-α and IL-6 (**a**) BMM were treated LPS (1μg/mL) for the indicated times. The concentration of TNF α and IL-6 in the culture media were measured by ELISA.* *p* < 0.01 *vs*. control, ^&^ *p* < 0.01 *vs*. 2 h, ^$^ *p* < 0.01 *vs*. 4 h, ^#^ *p* < 0.01 *vs*. 12 h. (**b**) BMM were pretreated Lut (12.5, 25 and 50 μM) 90 min, after incubated with or without LPS (1 μg/mL) for 12 h. The concentration of TNF α and IL-6 in the culture media were measured by ELISA. The results shown are the means ± S.D. of triplicate determinations. * *p* < 0.01 *vs*. control, ^&^ *p* < 0.01 *vs.* Lut (0 μM), ^$^ *p* < 0.01 *vs.* Lut (12.5 μM), ^#^ *p* < 0.01 *vs.* Lut (25 μM).

Our results showed that the maximum expression of TNF-α and IL-6 occurred 12 h after the LPS stimulation of BMM. Next, to assess the optimal concentration of Lut for the suppression of TNF-α and IL-6 expression, BMM were pretreated with Lut (12.5, 25 and 50 μM) for 1.5 h, and then stimulated with LPS (1 μg/mL) for 12 h. The concentrations of TNF-α and IL-6 in the culture media were measured by enzyme-linked immunosorbent assay (ELISA). As shown in Figure 3, Lut inhibited the production of TNF-α and IL-6 in a dose-dependent manner. The maximal inhibition of TNF-α and IL-6 production by Lut was observed at a concentration of 50 μM. The ability of Lut to suppress TNF-α and IL-6 production in this study is consistent with prior studies that suggested Lut can inhibit LPS-induced TNF-α and IL-6 production in macrophages [34].

### 2.2. Lut Promotes the Degradation of TNF-α and IL-6 mRNAs

**Figure 4:**
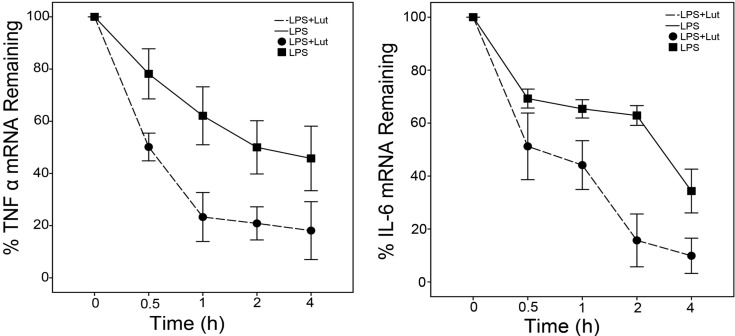
Lut promotes the degradation of TNF-α and IL-6 mRNAs BMM were pretreated with Lut (50 μM) or DMSO for 90 min, and then treated with LPS (1 μg/mL) for 90 min, after incubated with actinomycin D (Act D, 5 μg/mL) and LPS (1 μg/mL) for the indicated times. TNF α and IL-6 mRNAs were analyzed by real-time PCR, normalized by β-actin, and plotted. The results shown are the mean ± S.D. of three independent experiments.

Previous studies discovered that mRNA stabilization played an important role in coordinating LPS-mediated inflammation [35]. Real-time PCR analysis was performed to determine the half lives of the TNF-α and IL-6 mRNAs in our experimental model. BMM were initially pretreated with or without Lut (50 μM) for 1.5 h, and then incubated with LPS (1 μg/mL) for 1.5 h to induce inflammatory gene expression. Next, BMM were treated with actinomycin D (Act D, to block transcription) and LPS (to stabilize mRNAs) for the indicated times. As shown in Figure 4, compared to the LPS group, Lut accelerated the rate of decay of the mRNAs of interest, as the half lives of the TNF-α and IL-6 mRNAs were significantly shorter in the Lut group. These results suggest that Lut inhibits the expression of TNF-α and IL-6 at least in part through promoting mRNA degradation. 

### 2.3. Lut Affects the mRNAs Stability of TNF-α and IL-6 via the p38/MK2/TTP Signaling Cascade

**Figure 5:**
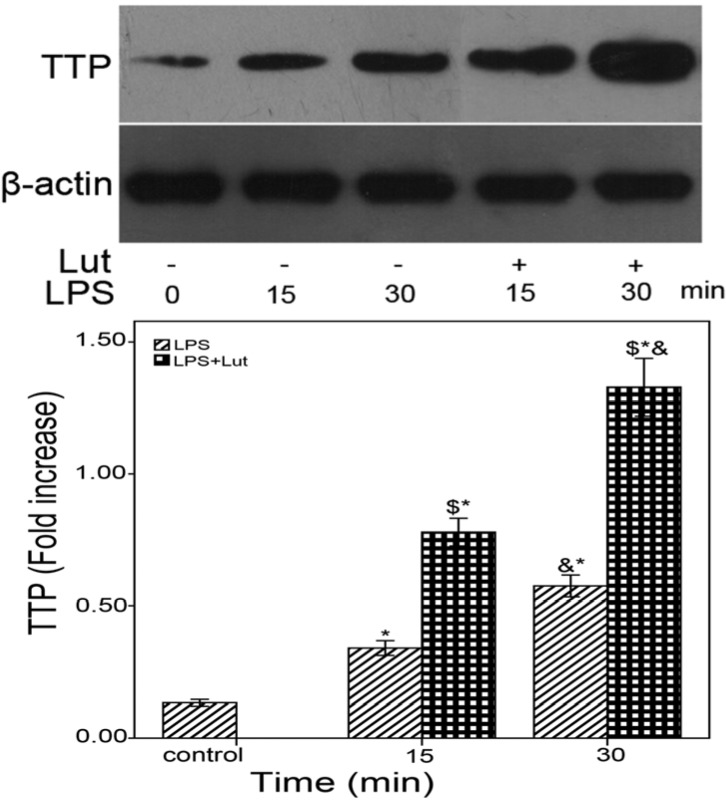
Lut affects TTP expression BMM were pretreated with Lut (50 μM) or DMSO for 90 min, after incubated with or without LPS (1 μg/mL) for the indicated times. The cell lysates were analyzed by the western blot method with antibodies TTP. * *p* < 0.01 *vs.* control, ^&^ *p* < 0.01 *vs.* 15 min, ^$^ *p* < 0.01 as (LPS + Lut group) *vs.* (LPS group).

Patil *et al*. has shown that TTP overexpression *in vivo* and *in vitro* attenuates LPS-induced inflammation [36]. Previous studies have also demonstrated that the decay of TNF-α and IL-6 mRNAs is regulated through the action of TTP [26,37]. Therefore, Western blots were performed to determine whether TTP protein expression is regulated by Lut. The cells were pretreated with or without Lut (50 μM) for 1.5 h and then incubated with LPS (1 μg/mL) for the indicated times. As shown in Figure 5, compared to the LPS group, Lut enhances TTP protein expression. Western blots have also been performed for another RNA binding protein HuR which has been identified to regulate TNF-α and IL-6 expression. The results showed that there was no significant difference between Lut group and LPS group on HuR protein expression.

**Figure 6:**
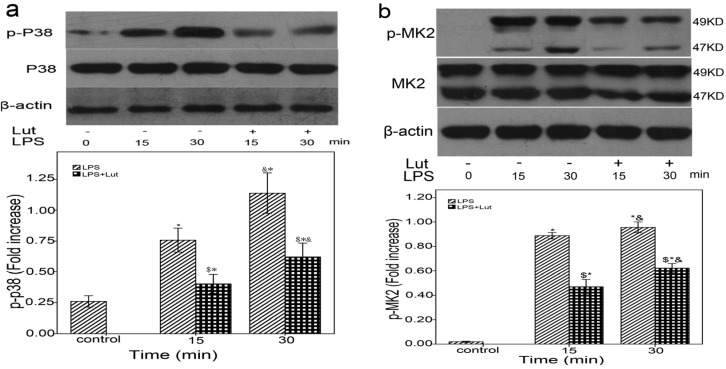
Lut affects p38 and MK2 activity a,b BMM were pretreated with Lut (50 μM) or DMSO for 90 min, after incubated with or without LPS(1 μg/mL) for the indicated times. The cell lysates were analyzed by the western blot method with antibodies p38, p-p38, MK2 and p-MK2. * *p* < 0.01 *vs.* control, ^&^ *p* < 0.01 *vs.* 15 min, ^$^ *p* < 0.01 as (LPS + Lut group) *vs.* (LPS group).

A previous study reported that TTP accumulation is reduced in wild-type macrophages compared with MK2-deficient macrophages upon LPS stimulation, indicating a role for MK2 in mediating LPS-induced TTP stabilization [38]. Another study reported that MK2 activation plays a crucial role in LPS-induced TTP modification and stabilization [39,40,41]. To examine whether the anti-inflammatory effect of Lut acts through p38/MK2-mediated regulation of TTP activity, western blots were carried out to analyze p38 and MK2 activity. As shown in Figure 6, compared to the LPS group, the phosphorylation levels of p38 and MK2 were suppressed in the Lut group after 15–30 min.

## 3. Experimental

### 3.1. Materials

Lut and LPS (*Escherichia coli* 055:B5) were obtained from Sigma-Aldrich (St Louis, MO, USA). Fetal bovine serum (FBS), Dulbecco’s modified Eagle medium (DMEM) and 0.25% trypsin were purchased from Gibco (Gaithersburg, MD, USA). Antibodies against phospho-p38, phospho-MK2, TTP, p38 and MK2 were purchased from Cell Signaling Technology (Danvers, MA, USA). ELISA kits for IL-6 and TNF-α were purchased from Western Tang (Shanghai, China). TRIzol reagent was obtained from Invitrogen(Carlsbad, CA, USA), the first-strand cDNA synthesis kit and SYBR Green Master Mix were purchased from(Applied Biosystems, Courtaboeuf, France).

### 3.2. BMM Cultures

Briefly, the tibia and femur were quickly excised and taken out after the Sprague-Dawley rats were sacrificed (Sprague-Dawley rats: 8–10 weeks old, male or female, 180 ± 20 g, provided by the Institute of Laboratory Animal of Xuzhou Medical College). All experimental protocols used in this study were approved by the ethical Committee in Xuzhou Medical College. Tibia and femur bone marrow of rats were obtained by flushing with DMEM. The bone marrows were cultured in DMEM with 20% heat-inactivated FBS and 30% L929 cell-conditioned medium at 37 °C in an atmosphere containing 5% CO_2_ for 5–7 days to simulate the differentiation and proliferation of BMM.

### 3.3. MTT Assay

The MTT assay was used to measure the viability of BMM. Differentiated BMM were digested, centrifuged, and inoculated in 96-well flat-bottomed plates. Each well contained 1 × 10^4^ BMM in suspension. After being cultured in DMEM with 20% heat-inactivated FBS and 30% L929 cell-conditioned medium for 3 h, cells were placed in serum-free media for another 1 h. Various concentrations of Lut (12.5, 25, 50, 100 μM) were added to BMM for 24 h. MTT solution (5 mg/mL) was added to each well. Following a 4-h incubation at 37 °C, the cell culture medium was removed and 100 μL of dimethyl sulfoxide (DMSO) was added to each well. The absorbance of each well was measured with an ELx 800 Universal Microplate Reader (Bio-Tek; Winooski, VT, USA) with the detection wavelength set at 570 nm. The viability of BMM in the Lut groups was expressed as a percentage of the viability of control BMM (which was considered to be 100%).

### 3.4. ELISA

BMM were pretreated with or without various concentrations of luteolin for 1.5 h before LPS (1 μg/mL) stimulation. Then, the ELISA was carried out according to the manufacturer’s instructions to determine the protein levels of TNF-α and IL-6 in the culture supernatants. 

### 3.5. RNA Isolation and Reverse Transcription

Total RNA were extracted from BMM using TRIzol reagent according to the manufacturer’s protocol. The concentration of total RNA was determined by spectrophotometry. RNA purity was confirmed by an optical density 260/280 nm ratio between 1.8 and 2.0. Total RNA (2 μg) were reverse-transcribed to cDNA at 70 °C for 5 min, and then cooled on ice. A first-strand cDNA synthesis kit was used at 42 °C for 60 min, followed by a 70 °C step for 15 min to terminate reaction.

### 3.6. Real-Time PCR

The mRNA levels of TNF-α, IL-6 and β-actin were measured by SYBR green-based real-time PCR. The real-time PCR reaction was performed in a total volume was 20 μL with SYBR green PCR Master Mix; 2 μL of cDNA sample was used as a template. Thermal cycling was performed with the following parameters: an activation step at 95 °C for 30 s, followed by 40 amplification cycles of denaturation at 95 °C for 30 s and annealing/extension at 60 °C for 2 min; fluorescence measurement took place at 72 °C. The fluorescence of the SYBR green dye was plotted as a function of PCR cycle number. In order to confirm the specificity of amplification, the PCR products from each primer pair were subjected to a melting curve analysis. The ΔCT values (Ct = cycle threshold value) of the target genes (TNF-α and IL-6) were calculated by subtracting the values of the experimental group from the housekeeping gene (β-actin) values. The 2-∆(∆Ct)method was used to calculate the relative expression of TNF α and IL-6 mRNA [36]. The primer sequences were designed using online tools as follows. Rat IL-6 forward, 5′-CTTCCAGCCAGTTGCCTTCTTG-3′, reverse, 5′-GGTCTGTTGTGGGTGGTATCCTC-3′; Rat TNFα forward, 5′-CCACCACGCTCTTCTGTCTACTG-3′, reverse, 5′-GGGCTACGGGCTTGTCACTC-3′, Rat β-actin forward, 5′-CCCATCTATGAGGGTTACGC-3′, reverse, 5′-TTTAATGTCACGCACGATTTC-3′.

### 3.7. Western Blot

BMM were harvested, washed in phosphate-buffered saline twice and lysed in cell lysis buffer (20 mM Tris, PH 7.5, 150 mM NaCl, 1% Triton×100, 1 mM PMSF, 1 mM EDTA, 2.5 mM sodium pyrophosphate) for 20 min. Lysed cells were centrifuged at 4 °C 12000g for 10 min, and the supernatant was collected. Protein concentration was determined by Bradford assay. Equal amounts of protein were separated by 12% SDS-PAGE and transferred onto polyvinylidene fluoride. The membranes were blocked for 2 h in 5% powdered milk in TBS containing 0.05% Tween 20 (TBS-T), followed by incubation with one of the following primary antibodies overnight at 4 °C: mouse anti TTP (1:1,000), rabbit anti-HuR (1:1,000), rabbit anti-phospho-MK2 (1:1,000), rabbit anti-MK2 (1:1,000), rabbit anti-phospho-p38 (1:1,000), mouse anti-p38 (1:1,000) and mouse anti-β-actin (1:1,000), with TBS-T containing 5% BSA. Membranes were washed in TBS-T and incubated with goat anti-rabbit and horse anti-mouse horseradish peroxidase (HRP)-conjugated secondary antibody (1:1,000). The membranes were washed again in TBS-T, and the binding of HRP-conjugated antibodies was detected by the ECL method according to the manufacturer’s instructions. Analysis of bands was done using the Image J software (NIH, Bethesda, MD, USA) and target protein band intensity values were normalized to β-actin values.

### 3.8. Statistical Analysis

Values are expressed as the mean ± SD. Statistical analysis was performed using SPSS 16.0 (Chicago, IL, USA). ANOVA with a post-hoc Bonferroni/Dunn test and an unpaired t-test was used to analyze the differences of the time intervals within the same treatment group and of the time intervals between the groups, respectively. A difference with a p value less than 0.05 was considered statistically significant.

## 4. Conclusions

In summary, our results show that Lut significantly ameliorates the LPS-induced inflammatory responses. This anti-inflammatory effect is partially mediated through the activation of the p38/MK2/TTP signaling cascade, leading to an increase in TTP, which targets the ARE-containing TNF-α and IL-6 mRNAs.
